# A new species of frog of the genus *Pristimantis* from Tingo María National Park, Huánuco Department, central Peru (Anura, Craugastoridae)

**DOI:** 10.3897/zookeys.610.8507

**Published:** 2016-08-11

**Authors:** Germán Chávez, Alessandro Catenazzi

**Affiliations:** 1 Centro de Ornitología y Biodiversidad (CORBIDI), Calle Santa Rita 105, Urb. Los Huertos de San Antonio, Surco, Lima, Peru; 2 Department of Zoology, Southern Illinois University, Carbondale, IL 62901, USA

**Keywords:** Amphibian, Andes, Craugastoridae, premontane forests, taxonomy, Anfibio, bosques premontanos, Craugastoridae, Andes, taxonomía

## Abstract

A new species of Craugastoridae frog encountered from 1000–1700 m in elevation in the premontane forests of the Peruvian central Andes is described. The new species is similar in appearance to many other species of *Pristimantis*, but is easily distinguishable from these species by having bright red coloration on the groin, posterior surface of thighs, and shanks. The new species is only known for two localities 27 km apart in the Huánuco Region.

## Abstract (es)

Describimos una nueva especie de rana de la familia Craugastoridae de los bosques premontanos de los Andes centrales peruanos, los especímenes fueron encontrados entre los 1000 – 1700 metros de elevación. Esta especie es similar en apariencia a muchas especies de *Pristimantis*, sin embargo es facilmente distinguible por tener ingles, superficie posterior de los muslos y de la tibia rojo brillante. La nueva especie es conocida solo de dos localidades en la Región Huánuco, ambas separadas por alrededor de 27 km.

## Introduction

Frogs of the genus *Pristimantis* (Craugastoridae) comprise one of the most striking, richest, and understudied groups in the Neotropics (Hedges et al. 2008; Padial and De la Riva 2009; Ortega–Andrade et al. 2015). Likewise, 131 species of *Pristimantis* are known to occur in Peru where they are distributed in many habitats on the western and eastern versants of the Andes (Frost 2016).

Although several species of Craugastoridae have been described from the eastern Andean slopes of central Peru over the last 15 years (Lehr et al. 2000; Lehr 2001; Lehr and Aguilar 2002; Lehr et al. 2002; Lehr and Aguilar 2003; Duellman and Hedges 2005; Boano et al. 2008; Duellman and Chaparro 2008; Lehr and Oroz 2012; Chávez et al. 2015), amphibian taxonomic research in one of main drainages of the area: the Upper Huallaga river has focused on species of Bufonidae, Hylidae, Centrolenidae and Dendrobatidae (Duellman and Toft 1979; Aichinger 1991, Castroviejo–Fisher et al. 2009) with only one Pristimantinae (Craugastoridae) species recently described from this region (Duellman and Hedges 2007).


 Tingo María National Park (TMNP) is a small protected area covering 4777 ha (SERNANP 2015) located in the Huallaga river basin on the eastern versant of the Peruvian central Andes. The landscape is dominated by a chain of small, isolated mountains commonly called “La Bella Durmiente” (“The sleeping beauty”), and belongs to lower montane forest (Gentry 1993). Few biological surveys have been conducted within this park, and its biological diversity is poorly known because terrorism and drug trafficking made the area inaccessible during the decades of the 80’s and 90’s. A rapid biological inventory was realized in 2014, 50 years after the creation by Peruvian law of TMNP, and resulted in an amphibian collection obtained by GC. Morphological analysis of collected specimens, and comparisons with similar species, led us to the discovery of a new species of frogs of the genus *Pristimantis*, which is described herein.

## Material and methods

Format for diagnosis and description of the new species follow those of Lynch and Duellman (1997). For systematic of Craugastoridae we follow Hedges et al. (2008), Pyron and Wiens (2011), and Padial et al. (2014). Specimens collected were sacrificed with a 20% benzocaine solution and fixed in 10% formalin, then stored in 70% ethanol and deposited in the herpetological collection at Centro de Ornitologia y Biodiversidad
(CORBIDI). The following variables were taken as described in Duellman and Lehr (2009) and were measured to the nearest 0.1 mm with digital calipers under a stereoscope: snout–vent length (SVL); eye–nostril distance (EN); head length (HL); head width (HW); interorbital distance (IOD); internarial distance (IND); tibia length (TL); foot length (FL); eye diameter (ED); upper eyelid width (EW).

Fingers and toes are numbered preaxially to postaxially from I–IV and I–V respectively. We determined comparative lengths of toes III and V by adpressing both toes against Toe IV; lengths of fingers I and II were determined by adpressing the fingers against each other. Specimens were sexed based on external sexual characteristics (e.g., presence of vocal sacs in males), all specimens were collected when they were calling, thus are considered adult males. Photographs were taken in the field and laboratory by GC, and used for descriptions of coloration in life and in preserved condition respectively. In addition to the type series of the new species, specimens examined are listed in Appendix I.

The advertisement calls of a chorus of males (CORBIDI 15563–68, 15577–78) were recorded at the type locality on 21 November 2014 (T_air_ = 23.0 °C; taken with a digital thermo hygrometer to the nearest 0.1 °C). A digital recorder (Marantz PMD661MK2) and unidirectional microphone (Sennheiser ME64) were used for field recording, and Raven Pro version 1.4 (Cornell Laboratory of Ornithology, Ithaca, NY) to analyze call variables. A total of 48 calls were analyzed. The following variables were measured from oscillograms: note, duration, and rate, interval between notes or calls, number of pulses, and presence of amplitude modulation. Variables measured from spectrograms included dominant frequency, and presence of frequency modulation or harmonics. Spectral parameters were calculated through fast Fourier transform (FFT) set at a length of 512 points (Hann window, 50% overlap). Averages are reported ± SD.

Genetic distances were estimated to confirm generic placement of the new species within *Pristimantis* through analysis of the non-coding 16S rRNA mitochondrial fragment. Tissues from two paratopotypes, CORBIDI 15563 and 15566, were used to obtain DNA sequences for the new species (deposited in GenBank; Appendix 2). Sequences of closely related, congeneric species were downloaded from GenBank (Appendix 2). Extraction, amplification, and sequencing of DNA followed standard protocols (Hedges et al. 2008; Catenazzi and Tito 2016). The 16Sar (forward) primer (5’-3’ sequence: CGCCTGTTTATCAAAAACAT) and the 16Sbr (reverse) primer (5’-3’ sequence: CCGGTCTGAACTCAGATCACGT) (Palumbi et al. 2002) were used; the following thermocycling conditions during the polymerase chain reaction (PCR) with a Veriti thermal cycler (Applied Biosystems) were employed: one cycle of 96 °C/3 min; 35 cycles of 95 °C/30 s, 55 °C/45 s, 72 °C/1.5 min; one cycle 72 °C/7 min. PCR products were purified with Exosap-IT (Affymetrix, Santa Clara, CA) and shipped to MCLAB (San Francisco, CA) for sequencing. Geneious R8, version 8.1.6 (Biomatters, http://www.geneious.com/) was used to align the sequences with the MAFFT, version 7.017 alignment program (Katoh and Standley 2013). Uncorrected p-distances (i.e., the proportion of nucleotide sites at which any two sequences are different) were estimated with the R package “ape” (Paradis et al. 2004).

## Results

### Pristimantis pulchridormientes sp. n.

Taxon classificationAnimaliaAnuraCraugastoridae

http://zoobank.org/4DCDA666-2217-48A0-9E6D-C1681544BDD5

Proposed standard English name: Sleeping beauty rain frog

Proposed standard Spanish name: Rana de lluvia de la Bella Durmiente

#### Holotype.

**Figure 1.**
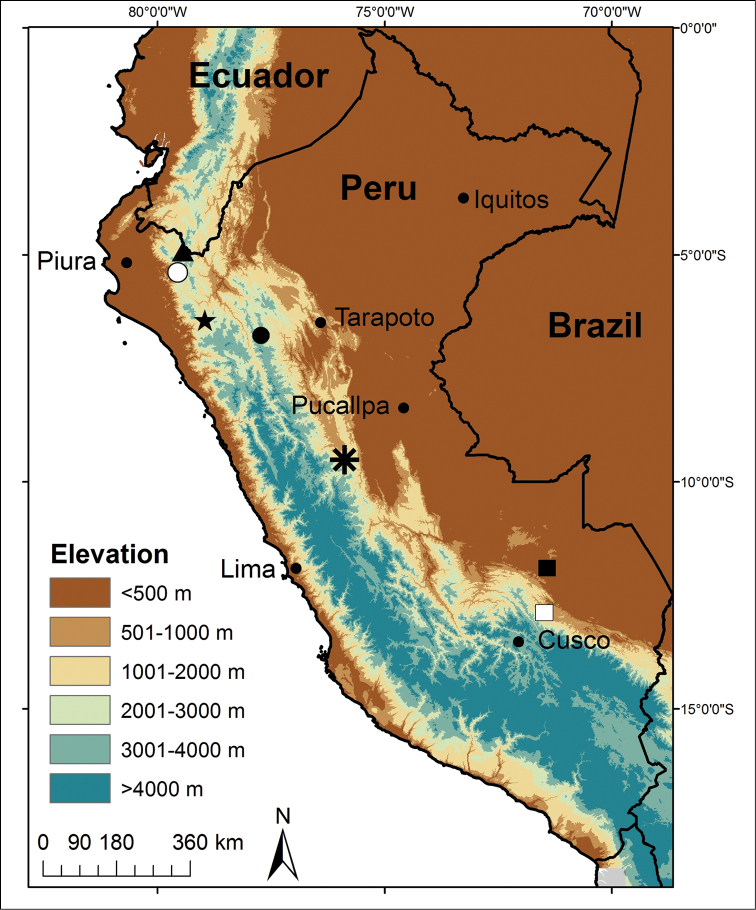
Map of Peru indicating the type locality of *Pristimantis pulchridormientes* sp. n. (asterisk), the two most closely related species according to analysis of genetic distances, *Pristimantis pluvialis* and *Pristimantis* sp. (white square; see text for analysis), and of other Peruvian species of *Pristimantis* with red shanks or thighs: *Pristimantis buccinator* (black square), *Pristimantis cajamarcensis* (black star), *Pristimantis ceuthospilus* and *Pristimantis rhodoplichus* (white circle), *Pristimantis coronatus* (triangle), *Pristimantis corrugatus* (black circle).

**Figure 2.**
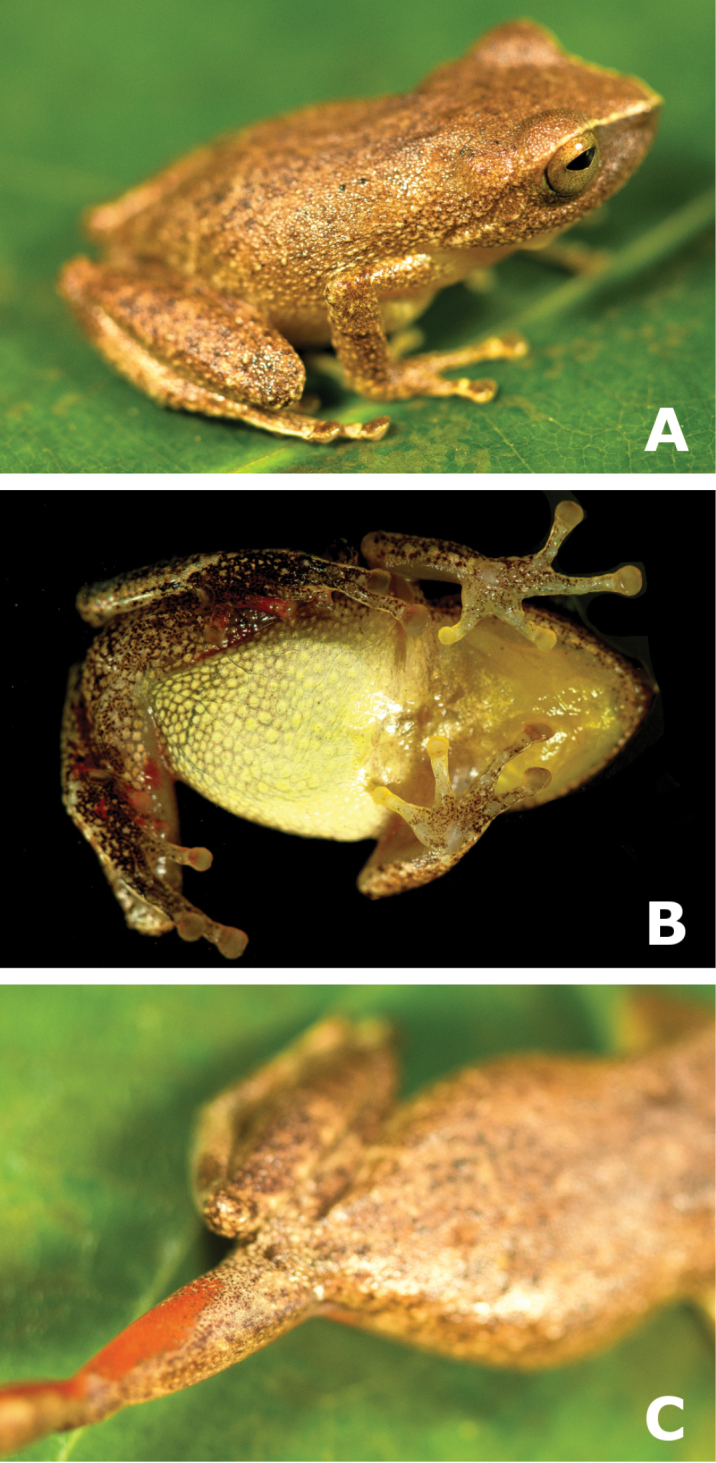
Dorsolateral and ventral views (**A, B**) of the holotype of *Pristimantis pulchridormientes* sp. n., male CORBIDI 15578, SVL = 21.9 mm, showing detail of (**C**) coloration on shanks and thighs. Photographs by G. Chávez.

**Figure 3.**
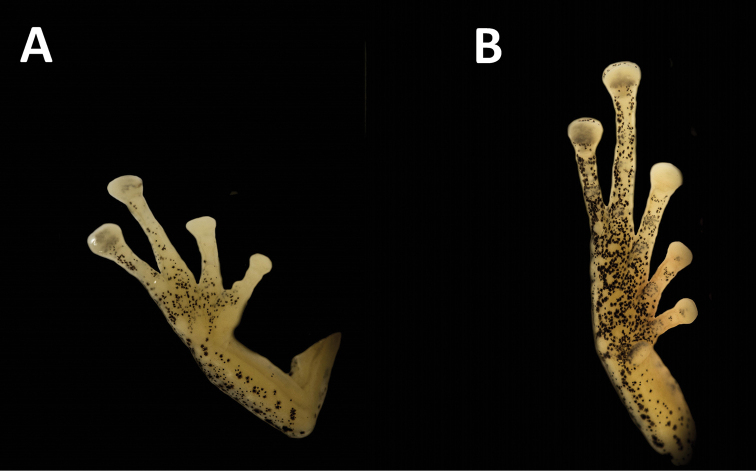
Ventral view of **A** hand (hand length – 8.5 mm) and **B** foot (foot length = 9.2 mm) of the holotype of *Pristimantis pulchridormientes* sp. n., (CORBIDI 15578). Photographs by Germán Chávez.

CORBIDI 15578 (Figures 1–3), an adult male collected by G. Chávez and D. Vásquez at Campamento La Garganta de la Bella, Tingo María National Park, (9°20'18.3"W, 76°0'7.4"S; 1095 m above sea level (asl), Provincia Leoncio Prado, Departamento Huánuco, Peru, on 21 November 2014.

**Figure 4.**
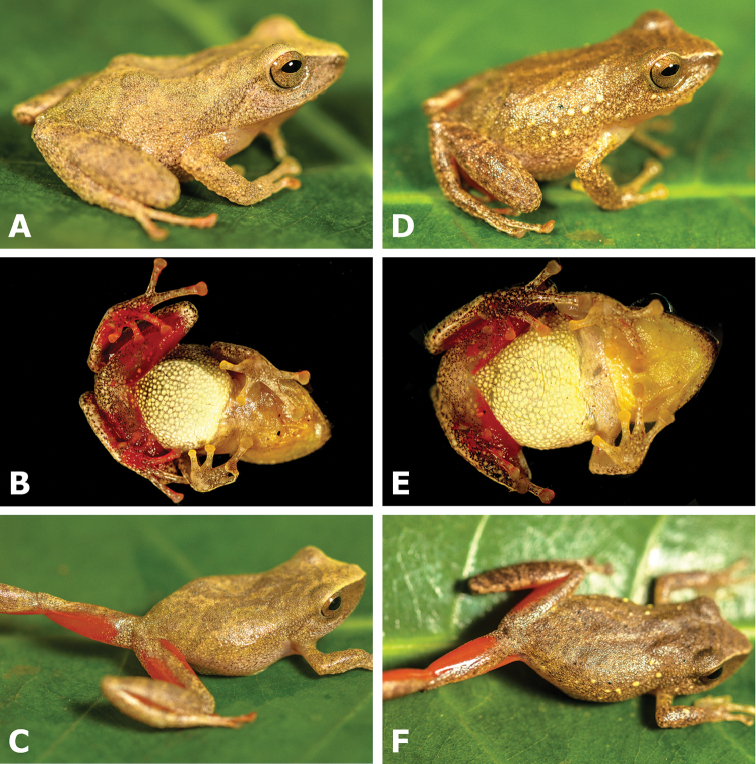
Dorsolateral and ventral views of two paratopotypes of *Pristimantis pulchridormientes* sp. n. showing detail of coloration on shanks and thighs. Male CORBIDI 15563 (**A–C**), SVL = 21.0 mm. Male CORBIDI 15565 (**D–F**), SVL = 21.5 mm. Photographs by G. Chávez.

Paratopotypes. Seven adult males (Fig. 4): CORBIDI 15563–68, 15577, collected along with the holotype.

Paratype. Adult male, CORBIDI 16606 collected at Sharco (9°35'58.6"W, 75°54'1.1’’ S; altitude 1700 m asl), Provincia Pachitea, Departamento Huánuco, Peru, on 28 November 2015 by Juan Carlos Chávez-Arribasplata.

#### Diagnosis.

The new species is distinguished by the following combination of characters: (1) skin on dorsum finely shagreen, that on venter areolate, discoidal fold absent, dorsolateral folds absent; (2) tympanic membrane and tympanic annulus distinct, weak supratympanic fold covering dorsal and posterior edges of tympanum, horizontal diameter of eye 3x the diameter of tympanum; (3) snout acuminate in dorsal view, truncated and posteroventrally inclined in lateral view, canthus rostralis weakly concave in dorsal view, angular in lateral view, loreal region concave, rostral papilla absent; (4) upper eyelid lacking tubercles, cranial crests absent; (5) dentigerous process of vomers absent; (6) males with vocal sacs and vocal slits, nuptial excrescences absent; (7) finger I and finger II of equal length, fingers II and III bearing rounded discs about 1.5 times wider than digits, finger IV bearing a rounded disc about twice as wide as its digit; (8) fingers with narrow lateral fringes; (9) antebrachial tubercle absent; (10) ulnar and tarsal tubercles absent (11) inner metatarsal tubercle oval twice as long as round outer metatarsal tubercle, low supernumerary plantar tubercles at the base of toes I, II, and III; (12) toes with narrow lateral fringes, webbing absent, toe V longer than toe III; (13) in life, males with dorsum creamy yellow or yellowish brown with dark blotches; canthal stripe creamy white extending to the orbits; throat yellow; belly creamy white; groins, posterior surfaces of thighs, and shanks bright red; iris cream with brown flecks; (14) SVL in adult males 19.1–21.9 mm; SVL in females unknown.

#### Comparisons.

*Pristimantis pulchridormientes* sp. n. is morphologically similar to *Pristimantis acuminatus*, *Pristimantis bromeliaceus*, *Pristimantis enigmaticus*, *Pristimantis lacrimosus*, *Pristimantis limoncochensis*, *Pristimantis mendax*, *Pristimantis olivaceus*, *Pristimantis omeviridis*, *Pristimantis padiali*, *Pristimantis pardalinus*, *Pristimantis pluvialis*, *Pristimantis pseudoacuminatus*, *Pristimantis rhodostichus*, *Pristimantis schultei*, and *Pristimantis tantanti* in having the head and body slightly compressed dorso–ventrally, but differs from all of them by having bright red coloration on groins, and on the posterior surfaces of thighs and shanks. Furthermore, *Pristimantis pulchridormientes* lacks a rostral papilla, which is present in *Pristimantis acuminatus*, *Pristimantis bromeliaceus*, *Pristimantis lacrimosus* (variable), *Pristimantis olivaceus*, *Pristimantis omeviridis*, *Pristimantis pardalinus*, *Pristimantis pluvialis*, *Pristimantis rhodostichus*, and *Pristimantis schultei*. Other species further differ by the following characters: *Pristimantis enigmaticus* has a tarsal fold (absent) and is lacking vocal slits (present); *Pristimantis limoncochensis* has a smooth dorsum (finely shagreen), and is lacking vocal slits (present), and a differentiated tympanic annulus and membrane (tympanic annulus and membrane distinct); *Pristimantis mendax* has a sigmoid inner tarsal fold (absent) and dorsal skin shagreen with scattered spicules (finely shagreen without spicules); *Pristimantis padiali* has an evident supratympanic fold (weakly evident), small dentigerous processes of vomers (absent), tubercles on ulnar and tarsal region (absent) and lacks vocal slits (present); *Pristimantis tantanti* has small dentigerous processes of vomers (absent), elongated ulnar tubercles (absent) and lacks tympanic annulus and membrane (present) and vocal slits (present).

Only eight other species of Peruvian *Pristimantis* have red coloration on groins and posterior surfaces of thighs: *Pristimantis buccinator*, *Pristimantis cajamarcencis*, *Pristimantis ceuthospilus*, *Pristimantis coronatus*, *Pristimantis corrugatus*, *Pristimantis lythrodes*, *Pristimantis rhodoplichus* and *Pristimantis sagittulus*. *Pristimantis pulchridormientes* can be differentiated from these species by having skin on dorsum finely shagreen (shagreen with pustules in *Pristimantis cajamarcencis*; shagreen with dermal ridges in *Pristimantis coronatus*; shagreen to finely corrugated in *Pristimantis lythrodes*; coarsely shagreen in *Pristimantis rhodoplichus*; shagreen with low tubercles in *Pristimantis sagittulus*), skin on venter areolate (smooth in *Pristimantis buccinator*), snout acuminate in dorsal view (rounded in *Pristimantis cajamarcencis*; subacuminate in *Pristimantis lythrodes* and *Pristimantis rhodoplichus*; acutely rounded in *Pristimantis sagittulus*), truncated and posteroventrally inclined in lateral view (acutely rounded in *Pristimantis ceuthospilus*; rounded in *Pristimantis coronatus and Pristimantis lythrodes*; acuminate in *Pristimantis sagittulus*), upper eyelids lacking tubercles (bearing small rounded tubercles in *Pristimantis rhodoplichus*, conical tubercles in *Pristimantis coronatus* and *Pristimantis corrugatus*), tympanic annulus not prominent (prominent in *Pristimantis buccinator*, *Pristimantis ceuthospilus*, *Pristimantis rhodoplichus*, *Pristimantis sagittulus*; absent in *Pristimantis coronatus*), supratympanic stripe absent (present in *Pristimantis cajamarcencis*), fingers I and II of equal lengths (finger I longer than finger II in *Pristimantis buccinator*; finger I shorter than finger II in *Pristimantis ceuthospilus*, *Pristimantis lythrodes* and *Pristimantis rhodoplichus*), ulnar tubercles absent (distinct conical ulnar tubercles in *Pristimantis corrugatus*), heels lacking tubercles (bearing small subconical tubercles in *Pristimantis rhodoplichus* and prominent conical tubercles in *Pristimantis corrugatus* and *Pristimantis sagittulus*).

The uncorrected genetic distances (Table 2) support the generic placement of the new species and its distinctiveness with respect to superficially similar species. According to these analyses, the most closely related species is *Pristimantis pluvialis* (Shepack et al. 2016), which despite sharing a similar body shape can easily be distinguished from *Pristimantis pulchridormients* by the presence of a rostral papilla, larger size, and coloration patterns.

The new species is also similar to the recently described *Pristimantis ardyae* (Reyes–Puig et al. 2013) from Ecuador in having red groins (red or orange in *Pristimantis ardyae*), but can be distinguished by the following characters (condition for *Pristimantis ardyae* in parentheses): upper eyelid lacking tubercles (bearing two small rounded tubercles), low ulnar tubercles present (absent), and iris cream with brown flecks (orange with fine black reticulations).

#### Description of the holotype.

An adult male (CORBIDI 15578) with a SVL of 21.9 mm, head as wide as long; snout subacuminate in dorsal view and truncated in lateral view, relatively short (eye–nostril distance 12% of SVL); canthus rostralis distinct in lateral view; loreal region concave; nostrils protuberant, directed anteriorly; interorbital area flat, broader than upper eyelid (upper eyelid width 59% of interorbital distance); cranial crests absent; upper eyelid lacking tubercles; tympanic membrane distinct, differentiated of surrounding skin; tympanic annulus distinct, round with weak supratympanic fold obscuring upper and posterodorsal edges of annulus (Fig. 2); tympanum diameter 31% of eye diameter; postrictal tubercles absent. Choanae small, rounded, not concealed by palatal shelf of maxillary; tongue longer than wide and granular. Skin texture on dorsum and flanks finely shagreen; dorsolateral folds absent; venter areolate; thoracic fold present, discoidal fold absent, cloacal sheath absent. Forearm slender; ulnar tubercles low, ulnar fold absent; radio–ulnar length 23% of SVL; fingers with narrow lateral fringes; relative lengths of fingers I ≤ II < IV < III; palmar tubercle bilobed, thenar tubercle oval (Fig. 3); subarticular tubercles round, prominent; supernumerary palmar tubercles present at the base of all fingers; disc cover finger I slightly expanded, those of fingers III and IV extensively expanded (Fig. 3), outer discs of fingers as wide as those of toes; discs covered with elliptical ventral pads defined by circummarginal grooves. Hind limbs slender; tibia length 50% of SVL; foot length 85% of tibia length; tarsal fold absent, tarsal tubercles low; heel lacking tubercles; toes with narrow lateral fringes; subarticular tubercles round, prominent; inner metatarsal tubercle oval, about 2.4 times the size of subconical outer tubercle; supernumerary plantar tubercles low at the base of all toes; discs covers slightly expanded; toes with defined pads; discs pads nearly elliptical; relative length of toes I < II < III < V < IV; tip of toe V reaching proximal border of distal subarticular tubercle IV; tip of toe II reaching distal border of medial subarticular tubercle of Toe IV.

#### Measurements and proportions of the holotype

**(in millimeters).** SVL = 21.9; HL = 8.5; HW = 8.5; ED = 2.7; EN = 2.5; TD = 0.5; IOD = 3.1; EW = 1.8; IND = 1.9; TL = 10.8; FL = 9.2; HL/SVL = 0.3; HW/SVL = 0.3; EW/IOD = 0.5; TL/SVL = 0.4; FL/SVL = 0.4; FL/TL = 0.8.

#### Coloration in life.

At night, dorsum, flanks, and dorsal surface of limbs are yellowish-brown with diagonal brown blotches and tiny brown flecks; dorsal surface of head of the same color and bearing a fine creamy yellow canthal stripe which extends to the medial portion of the upper eyelids. Throat yellow, chest and belly are creamy-white with tiny dark flecks; ventral surface of hands, and exterior portion of the ventral surface of foots yellowish-brown; groins, posterior surface of thighs, posterior surface of shanks, and inner portion of the ventral surface of hands bright red. Anterior surface of thighs are pinkish-gray with irregular red blotches. Iris golden with fine dark flecks. In daytime, yellowish-brown coloration turns into pale yellow.

#### Coloration in preservative.

As described above, but yellowish-brown coloration turns creamy-yellow with tiny dark flecks on dorsum, limbs and ventral surfaces of hands and feet; red coloration turned pinkish-white, and venter creamy-yellow; iris gray.

#### Advertisement call.

**Figure 5.**
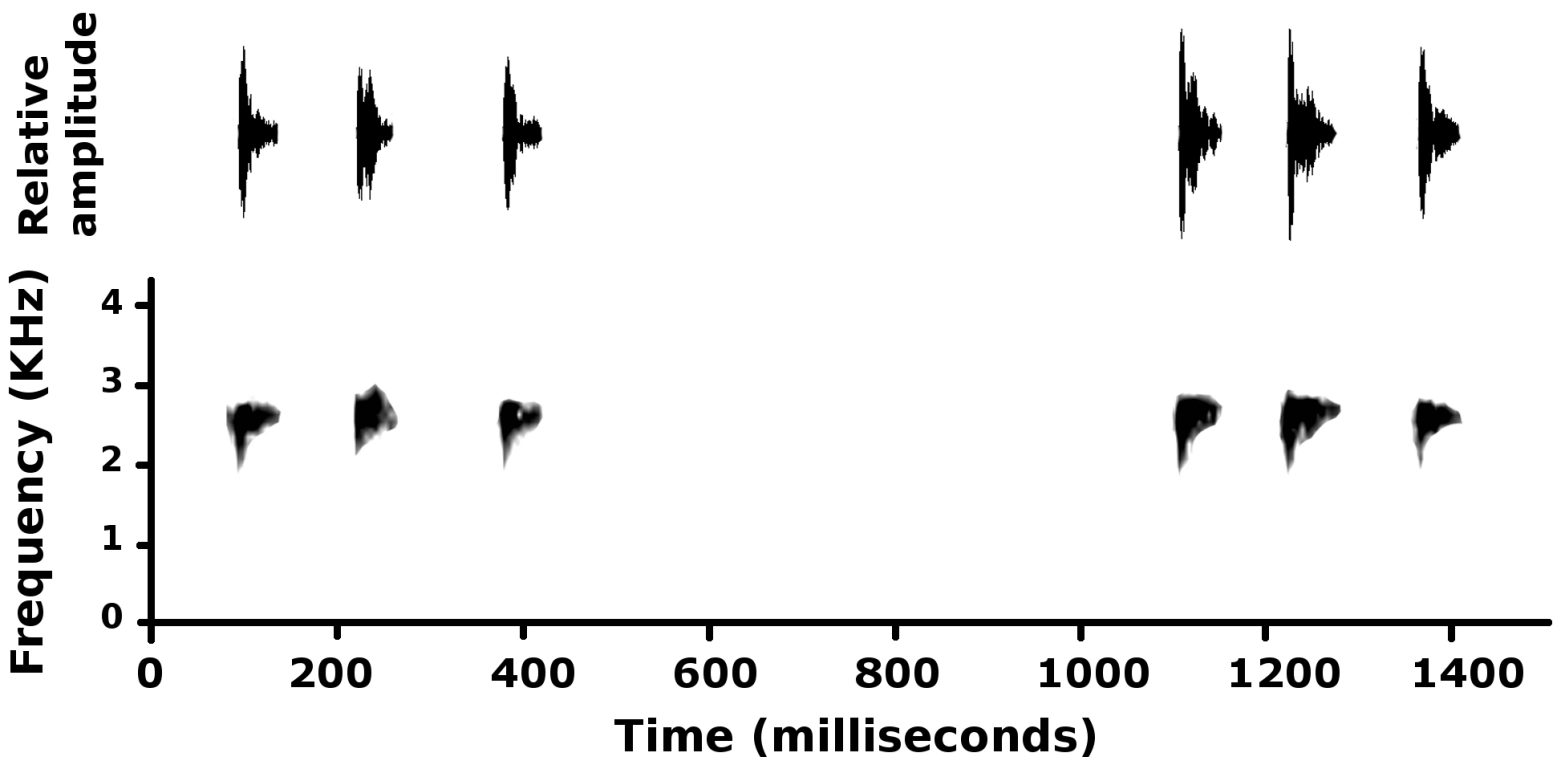
Oscillogram (above) and spectrogram and of the advertisement call of an uncollected male of *Pristimantis pulchridormientes* sp. n., recorded at the type locality on 21 November 2014 (T_air_ = 23.0 °C).

A chorus of several males (CORBIDI 15563–68, 15577–78) was recorded at a distance of 1 meter from the microphone; thus the description refers to such context. The general structure of calls in this chorus (2'45’’ recording) is that calls include a variable number of single–pulse notes (Fig. 5). It is possible, considering advertisement calls in similar *Pristimantis* species, that males emit simpler vocalizations, i.e., single notes separated by longer durations, outside of choruses. Males in the chorus produced calls with 3.3 ± 0.7 notes (range 2–5 notes). Note duration averaged 45.9 ± 12.6 ms (range 31–75 ms). Fundamental frequency averaged 2763 ± 133 ms (range 2531–3094 Hz) and did not vary within or among notes; likely the main source of variation in fundamental frequency was among individuals. Within single notes, much of the energy was concentrated in the first half of the note.

#### Variation.

Measurements and proportions of the specimens examined are given in Table 1. Dorsal coloration pattern is paler in CORBIDI 15563–64, 15568 than the holotype. Specimen CORBIDI 15565 (Figure 4, D–F) has a minute dorsolateral yellow spot. Specimens CORBIDI 15563–66 have a darker yellow throat.

**Table 1.** Measurements and morphological proportions of *Pristimantis pulchridormientes* sp. n. Range is followed by mean value and standard deviation in parenthesis (n = 9 adult males).

Snout-vent length (SVL)	19.1–21.9 (20.5 ± 0.8)
Head length (HL)	7.0–8.5 (7.8 ± 0.5)
Head width (HW)	7.2–8.5 (7.8 ± 0.5)
Upper-eyelid width (EW)	1.8–1.9 (1.8 ± 0.1)
Interorbital distance (IOD)	2.2–3.1 (2.8 ± 0.3)
Eye diameter (ED)	2.3–2.7 (2.6 ± 0.1)
Eye-nostril distance (EN)	2.2–2.5 (2.3 ± 0.1)
Internarial distance (IND)	1.6–1.9 (1.7 ± 0.1)
Tibia length (TL)	9.6–10.8 (10.3 ± 0.4)
Foot length (FL)	7.7 – 9.2 (8.3 ± 0.5)
HL/SVL	0.3–0.4 (0.3 ± 0.1)
HW/SVL	0.3–0.4 (0.3 ± 0.1)
FL/SVL	0.3–0.4 (0.3 ± 0.1)
EN/SVL	0.1 (0.1 ± 0.0)
FL/TL	0.7–0.8 (0.8 ± 0.0)
EW/IOD	0.5–0.8 (0.6 ± 0.0)

#### Etymology.

**Figure 6.**
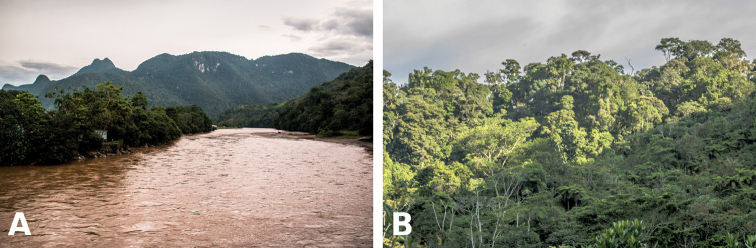
Habitat of *Pristimantis pulchridormientes* sp. n. in Tingo María National Park: **A** panoramic view of the Bella Durmiente (Sleeping Beauty) chain of mountains , and **B** premontane forest at the type locality. Photographs by G. Chávez.

The name is composed of two words in Latin, “pulcher” which means beautiful, and “dormientes” = sleeping, in reference to the chain of mountains located within Tingo María National Park, above the city of Tingo Maria, locally known as Sleeping Beauty (Bella Durmiente), because it looks like a sleeping reclined woman (Figure 6A).

#### Distribution and natural history.

*Pristimantis pulchridormientes* is known from two localities (Fig. 1), Garganta de la Bella, the highest point (1095 m asl) along the trail inside Tingo Maria NP, and Sharco (1700 m asl), approximately 27 km south of the type locality (by airline). Male specimens CORBIDI 15563–68, 15577–78 were collected at the beginning of the rainy season, calling at night, perched on leaves 2 meters above ground in the primary montane forest. Although bromeliads were present, individuals were not observed using these plants. Other vegetation included trees *Cecropia* spp. and *Cedrella* spp., bamboo patches, ferns, epiphyte plants and lichens (Figure 6B). The ground was covered with leaf litter and rocks. Sympatric species include the amphibians *Pristimantis diadematus*, *Pristimantis mendax*, *Pristimantis peruvianus*, *Pristimantis ockendeni*, and *Phyllomedusa camba*, and the reptiles *Anolis fuscoauratus*, *Copeoglossum nigropunctatum*, *Phrynonax polylepis*, and *Spillotes sulphureus*. One specimen (CORBIDI 16606) was collected calling at night on a leaf 2 m above the ground in a patch of a secondary forest, the habitat strongly affected by human activities such as cattle grazing and orange plantations. The vegetation consisted of trees of *Cecropia* spp., *Ficus* spp., and the ground was covered by herbs and bushes. Sympatric amphibians include *Pristimantis ockendeni* and Scinax aff. ruber.

## Discussion

The new species is not assigned to any taxonomic group despite the presence of morphological characters (i.e. head and body dorsoventrally compressed; skin on dorsum finely shagreen and that on venter areolate) suggesting a possible inclusion in the *Pristimantis lacrimosus* group. Group assignment is currently avoided because of the unclear taxonomic status of the *Pristimantis lacrimosus* group, as shown by recent phylogenetic studies (Padial et al. 2014; Ortega–Andrade et al. 2015). Further hindering group assignments among frogs of the genus *Pristimantis* are high species diversity (Hedges et al., 2008), small genetic distances (i.e. *Pristimantis acuminatus* complex; Ortega–Andrade et al. 2015), and limitations of morphological characters in defining species groups (Elmer and Cannatella 2008; Hedges et al. 2008; Padial and De la Riva 2009). Furthermore, phylogenetic relationships proposed by Hedges et al. (2008) for species in the putative *Pristimantis lacrimosus* group are not confirmed by recent studies, such as the strongly supported phylogeny published by Padial et al. (2014). In fact, Hedges et al. (2008) placed *Pristimantis acuminatus* outside of the *Pristimantis lacrimosus* group, but Padial et al. (2014) showed that this species is closely related to species of the putative *Pristimantis lacrimosus* group, evidencing the complicated taxonomy of this clade.

The analysis of genetic distances (uncorrected p-distances) shows that *Pristimantis pulchridormientes* sp. n. is closely related to both *Pristimantis pluvialis* and an undescribed species (*Pristimantis* sp.) from southeastern Peru (Table 2), which both show clear morphological differences with *Pristimantis pulchridormientes* (Shepack et al. 2016). In contrast, other species such as *Pristimantis acuminatus*, *Pristimantis bromeliaceus*, and *Pristimantis omeviridis*, show higher genetic distances exceeding 10%.

**Table 2.** Uncorrected p-distances of the mitochondrial 16S rRNA gene. Comparisons between *Pristimantis pulchridormientes* and the three taxa with lowest p-distances are indicated in bold.

	*Pristimantis acuminatus* (MC11555)	*Pristimantis boulengeri* (MAV257)	*Pristimantis bromeliaceus* (KU291702)	*Pristimantis cf. mendax* (MTD45080)	*Pristimantis dorsopictus* (MHUAA7638)	*Pristimantis galdi* (QCAZ32368)	*Pristimantis mindo* (MZUTI1382)	*Pristimantis mindo* (MZUTI1756)	*Pristimantis moro* (AJC1753)	*Pristimantis moro* (AJC1860)	*Pristimantis omeviridis* (QCAZ19664)	*Pristimantis pluvialis* (CORBIDI11862)	*Pristimantis pluvialis* (CORBIDI16695)	*Pristimantis pulchridormientes* (CORBIDI 15563)	*Pristimantis pulchridormientes* (CORBIDI 15566)	*Pristimantis ridens* (AJC1778)	*Pristimantis* sp. (CORBIDI 17473)	*Pristimantis* sp. (ROM43978)	*Pristimantis subsigillatus* (MECN10117)
*Pristimantis acuminatus* (MC11555)	0.00																		
*Pristimantis boulengeri* (MAV257)	0.05	0.00																	
*Pristimantis bromeliaceus* (KU291702)	0.14	0.13	0.00																
Pristimantis cf. mendax (MTD45080)	0.14	0.13	0.00	0.00															
*Pristimantis dorsopictus* (MHUAA7638)	0.15	0.13	0.11	0.11	0.00														
*Pristimantis galdi* (QCAZ32368)	0.11	0.11	0.10	0.10	0.08	0.00													
*Pristimantis mindo* (MZUTI1382)	0.12	0.11	0.09	0.09	0.08	0.06	0.00												
*Pristimantis mindo* (MZUTI1756)	0.12	0.12	0.09	0.09	0.08	0.06	0.00	0.00											
*Pristimantis moro* (AJC1753)	0.11	0.10	0.09	0.10	0.09	0.09	0.08	0.07	0.00										
*Pristimantis moro* (AJC1860)	0.11	0.10	0.09	0.09	0.09	0.09	0.07	0.07	0.05	0.00									
*Pristimantis omeviridis* (QCAZ19664)	0.14	0.13	0.12	0.12	0.12	0.11	0.12	0.11	0.10	0.10	0.00								
*Pristimantis pluvialis* (CORBIDI11862)	0.14	0.14	0.11	0.11	0.11	0.11	0.09	0.09	0.11	0.09	0.13	0.00							
*Pristimantis pluvialis* (CORBIDI16695)	0.14	0.14	0.11	0.11	0.11	0.11	0.09	0.09	0.11	0.09	0.13	0.00	0.00						
*Pristimantis pulchridormientes* (CORBIDI 15563)	0.18	0.17	0.13	0.12	0.13	0.12	0.11	0.10	0.10	0.10	0.14	**0.07**	**0.07**	0.00					
*Pristimantis pulchridormientes* (CORBIDI 15566)	0.18	0.17	0.13	0.12	0.13	0.12	0.11	0.10	0.10	0.10	0.14	**0.07**	**0.07**	0.00	0.00				
*Pristimantis ridens* (AJC1778)	0.15	0.13	0.14	0.14	0.13	0.16	0.13	0.13	0.14	0.13	0.15	0.15	0.15	0.16	0.16	0.00			
*Pristimantis* sp. (CORBIDI 17473)	0.14	0.14	0.11	0.11	0.10	0.09	0.08	0.08	0.10	0.08	0.12	0.06	0.06	**0.07**	**0.07**	0.15	0.00		
*Pristimantis* sp. (ROM43978)	0.14	0.13	0.12	0.12	0.11	0.10	0.09	0.08	0.10	0.10	0.13	0.07	0.07	**0.08**	**0.08**	0.17	0.06	0.00	
*Pristimantis subsigillatus* (MECN10117)	0.13	0.13	0.11	0.11	0.09	0.10	0.05	0.05	0.09	0.09	0.12	0.10	0.10	0.12	0.12	0.15	0.09	0.09	0.00

At both localities the new species was found in arboreal microhabitats, frequently calling perched on leaves 2 m above the ground. The only other species sharing a similar microhabitat and presumably ecological niche (also nocturnal) was Pristimantis aff. mendax, but this frog was not found in sympatry with *Pristimantis pulchridormientes*, because its altitudinal range of 100–900 m asl does not overlap with the range of *Pristimantis pulchridormientes* from 1095–1700 m asl.

With only two known localities, it is difficult to predict the potential distribution range of this species. Although the type locality had a large population and was located inside a protected area, the locality where paratype CORBIDI 16606 was collected had a very fragmented habitat surrounded by orange plantations and corn cropland. Considering that the upper Huallaga drainage is highly disturbed by agriculture activities which fragments the submontane and montane forests (Catenazzi and von May 2014), the distribution of *Pristimantis pulchridormientes* is considered to be highly fragmented with the only known protected population living on an isolated slope of Tingo Maria NP. On the basis of our limited knowledge of its distribution, and according to the IUCN Red List guidelines (2016) we recommend the species be placed in the Data Deficient category of the IUCN Red List of Threatened species.

## Supplementary Material

XML Treatment for Pristimantis pulchridormientes

## Appendix 1

### Specimens examined

*Pristimantis acuminatus*.– **PERU: Amazonas**: Provincia Condorcanqui: CORBIDI 11388, 11403, Quebrada Kampankis, 4°02'35.1"S, 77°32'28.3"W, 325 m. **Loreto**: Provincia Loreto: CORBIDI 1128, San Jacinto, 2°19'51.0"S, 75°51'49.3"W, 160 m; CORBIDI 1531, 4720, 4769, Andoas, 2°42'15.6"S, 76°18'46.2"W, 273 m; Provincia Requena: CORBIDI 2204, Sierra del Divisor, 6°55'07.4"S, 73°50'43.0”, 205 m.

*Pristimantis bromeliaceus*.– **PERU: Amazonas**: Provincia Bagua: CORBIDI 0778, Chonza Alta, 5°35'18.5"S, 78°21'02.2"W, 2047 m; CORBIDI 1972–73, 2037–39, Catarata de Cañopite, 5°36'57.8"S, 78°19'48.6"W, 2335 m; CORBIDI 5547, 5550, 5557, 5560–61, 5566–67, 5570, 5580, Área Conservación Privada Copallín, 5°36'59.7"S, 78°20'33.9"W, 2046 m. **Ayacucho**: Provincia La Mar: CORBIDI 6934, 6953–54, Chiquintirca – San Antonio road, 12°57'26.3"S, 73°37'03.4"W, 2305 m. **Cajamarca**: Provincia San Ignacio, CORBIDI 14820, El Chaupe, 5°17'48.0"S, 72°02'09.7"W, 1891 m. **Pasco**: Provincia Oxapampa: CORBIDI 3859, Comunidad Campesina Chacos, 10°33'06.2"S, 75°20'18.1"W, 2901 m; CORBIDI 11520–21, 11562, 11567, Bosque de Sho´llet, 10°37'49.3"S, 75°16'58.4"W, 2181 m **San Martin**: Provincia Moyobamba: CORBIDI 3143–44, 3176, Paitoja 6°21'09.8"S, 77°04'03.6"W, 1731 m; Provincia Rioja: CORBIDI 0510–12, 0516–17, Abra Patricia, 5°41'45.5"S, 77°47'37.2"W, 2189 m.

*Pristimantis buccinator*.– **PERU: Cusco**: Provincia La Convención: CORBIDI 12479, Mipaya 11°34'46.5"S, 73°10'17.1"W, 377 m. **Loreto**: Provincia Requena: CORBIDI 3911–12, 3923, 3446, 3953, 3960, Sierra del Divisor 6°12'49.0"S, 73°14'21.0"W, 500 m.

*Pristimantis cajamarcencis*.– **PERU: Cajamarca**: Provincia San Ignacio: CORBIDI 14811, 14816, 14819, 14823, 14836, 14837, 14838, El chaupe 5°17'48.0"S, 72°02'09.7"W, 1891 m.

*Pristimantis ceuthospilus*.– **PERU: Piura**: Provincia Huancabamba: CORBIDI 4211, Chigña alta 5°35'01.6"S, 79°40'04.1"W, 715 m. **Lambayeque**: Provincia Lambayeque: CORBIDI 4295–99, 4230–46, Quebrada Palacios, Distrito Salas 6°01'15.5"S, 79°32'16.3"W, 1082 m.

*Pristimantis corrugatus*.– **PERU: Amazonas**: Provincia Chachapoyas: CORBIDI 10897–904, Bosque de palmeras de Ocol, Molinopampa, 6°16'03.7"S, 75°35'06.7"W, 2566 m; Provincia Rodríguez de Mendoza: CORBIDI 12877–82, 12884, La Colpa 6°23'37.0"S, 77°13'42.6"W, 2347 m. **San Martin**: Provincia Mariscal Cáceres: CORBIDI 11018, 11024–11028, 11030–39, Quintecocha 6°51'33.0"S, 77°42'14.7"W, 3119 m.

*Pristimantis lacrimosus*.– **PERU: Loreto**: Provincia Requena: CORBIDI 3941, Sierra del Divisor, 6°12'49.0"S, 73°14'21.0"W, 500 m, CORBIDI 12133–38; Rio Tapiche, 5°38'07.7"S, 73°55´25.3"W, 121 m; Provincia Putumayo: CORBIDI 5894, 5899, 5903, Campamento Piedras, 2°47'33.9"S, 72°55'00.6"W, 156 m.

*Pristimantis mendax*.– **PERU: Cusco**: Provincia La Convención: CORBIDI 8218, 8255, 8596–97, 8600–01, 8603, 8608, 8610, Alto Shimá, 12°32'32.5"S, 73°07´59.9"W, 1592 m. **Huánuco**: Provincia Huánuco: CORBIDI 14925, 14940–42, Santa Clara, 9°37'46.3"S, 75°49´49.8"W, 1085 m; CORBIDI 14992–93, Quebrada Mallgotingo, 9°36'48.2"S, 75°57´15.3"W, 1318 m; Provincia Leoncio Prado: CORBIDI 15494–95, 15499, 15500, 15520, 15553, Campamento La Quinceañera, Parque Nacional Tingo María, 9°22'16.8"S, 75°59'13.8"W, 1124 m; Provincia Pachitea: CORBIDI 13345–13346, Chaglla, 9°36'08.4"S, 75°54'10.0"W, 883 m; Provincia Puerto Inca: CORBIDI 13945–46, 14434–35, Campamento Peligroso, Serranía del Sira, 9°25'34.2"S, 74°44'06.6"W, 1525 m.

*Pristimantis olivaceus*.– **PERU: Cusco**: Provincia La Convencion: CORBIDI 8086, 10530, Comunidad Nativa Chokoriari, 11°57'25.0"S, 72°56'27.5"W, 434 m; CORBIDI 8296, 9765–66, Comunidad Nativa Puyentimari, 12°11'18.7"S, 73°0´03.3"W, 725 m; CORBIDI 10260, Kinteroni, 11°30'46.3"S, 73°15´06.3"W, 447 m. **Madre de Dios**: Provincia Tambopata: CORBIDI 5238, Lago Tres Chimbadas, 12°47'11.0"S, 69°12'21.1"W, 175 m; CORBIDI 13493–94, El Parador, 12°58'47.0"S, 70°14'06.2"W, 240 m.

*Pristimantis rhodoplichus*.– **PERU: Piura**: Provincia Ayabaca: CORBIDI 0448–49, Bosque de Cuyas, 4°40'01.0"S, 79°34'25.0"W, 2673 m.

*Pristimantis rhodostichus*.– **PERU: Loreto**: Provincia Datem del Marañon: CORBIDI 11430, Cabecera del Wee, Distrito Manseriche, 4°12'14.8"S, 77°31'47.2"W, 1435 m. **San Martin**: Provincia Mariscal Caceres: CORBIDI 0667–76, 0678–80, Laguna Negra 6°53'29.3"S, 77°23'18.3"W, 1788 m; Provincia Picota: CORBIDI 8867, 9933, Puesto de control 16, Parque Nacional Cordillera Azul 7°04'08.9"S, 76°00'55.2"W, 1122 m.

*Pristimantis schultei*.– **PERU: Amazonas**: Provincia Luya: CORBIDI 0368, Área de Conservación Privada Huiquilla, 6°22'45.0"S, 77°58´42.0"W, 2935 m; CORBIDI 12349–63, 12365–66, 12370–71, Distrito Lonya Chico, 6°14'01.7"S, 78°06'78.3"W, 2826 m; Provincia Bongará: CORBIDI 0452–62, Yuramarca, 6°02'16.7"S, 75°51'05.5"W, 2857 m; Provincia Rodríguez de Mendoza: CORBIDI 11689, 11693–96, 11701, 11708, 11725–33, 11737–38, 11740–41, Quebrada Salas (Distrito Vista Alegre), 6°06'42.9"S, 77°26'24.0"W, 2575 m; CORBIDI 12870–75, 12883, La Colpa 6°23'37.0"S, 77°13'42.6"W, 2347 m. **San Martin**: Provincia Mariscal Cáceres: CORBIDI 0597, 0603–04, 0606–07, 0612, 0614, 0616, Laurel, 6°41'00.4"S, 77°41'39.9"W, 2799 m; CORBIDI 15054, 15056, 15065–66, 15071, 15106–09, 15123, Albazo, 6°42'59.4"S, 77°40'27.5"W, 2404 m.

## Appendix 2

### Gene sequences for molecular analyses

**Table:** GenBank accession numbers for the taxa and genes sampled in this study. ^1^*Pristimantis* sp. (ROM 43978) is treated herein as *Pristimantis* sp. following Padial et al. (2014).

Taxon	Voucher Nbr.	16S
*Pristimantis acuminatus*	MC 11555	DQ195448
*Pristimantis bromeliaceus*	KU 291702	EF493351
*Pristimantis boulengeri*	MAV 257	DQ195452
Pristimantis cf. mendax	MTD45080	EU186659
*Pristimantis dorsopictus*	MHUAA 7638	KP082874
*Pristimantis galdi*	QCAZ 32368	EU186670
*Pristimantis mindo*	MZUTI 1382	KF801584
*Pristimantis mindo*	MZUTI 1756	KF801581
*Pristimantis moro*	AJC 1860	JN991454
*Pristimantis moro*	AJC 1753	JN991453
*Pristimantis omeviridis*	QCAZ19664	EU130579
*Pristimantis pluvialis*	CORBIDI 11862	KX155577
*Pristimantis pluvialis*	CORBIDI 16695	KX155578
*Pristimantis pulchridormientes* sp. n.	CORBIDI 15563	KX664106
*Pristimantis pulchridormientes* sp. n.	CORBIDI 15566	KX664107
*Pristimantis ridens*	AJC 1778	KR863320
*Pristimantis* sp. 1	CORBIDI 17473	KX155579
*Pristimantis* sp. 2	ROM 43978	EU186678
*Pristimantis subsigillatus*	MECN 10117	KF801580
